# An Overview of Systemic Health Factors Related to Rapid Oral Health Deterioration among Older People

**DOI:** 10.3390/jcm12134306

**Published:** 2023-06-27

**Authors:** Gert-Jan van der Putten, Cees de Baat

**Affiliations:** 1 Orpea Dagelijks Leven, 7327 AA Apeldoorn, The Netherlands; 2 Department of Dentistry, Radboud University Nijmegen Medical Centre, 6525 GA Nijmegen, The Netherlands; 3 Fresh Unieke Mondzorg, 2411 NT Bodegraven, The Netherlands

**Keywords:** oral health care, older people, multimorbidity, polypharmacy, frailty, sarcopenia, disability, care dependency

## Abstract

The oral health of older individuals can be negatively impacted by various systemic health factors, leading to rapid oral health deterioration. This paper aims to present an overview of the published evidence on systemic health factors that contribute to rapid oral health deterioration in older individuals, and to explore the implications of these factors for both general healthcare and oral healthcare provision. Older people are at risk of experiencing adverse reactions to medications due to multimorbidity, polypharmacy, and changes in pharmacokinetics and pharmacodynamics. Hyposalivation, a significant side effect of some medications, can be induced by both the type and number of medications used. Frailty, disability, sarcopenia, care dependency, and limited access to professional oral healthcare can also compromise the oral health of older people. To prevent rapid oral health deterioration, a comprehensive approach is required that involves effective communication between oral healthcare providers, other healthcare providers, and informal caregivers. Oral healthcare providers have a responsibility to advocate for the importance of maintaining adequate oral health and to raise awareness of the serious consequences of weakened oral health. By doing so, we can prevent weakened oral health from becoming a geriatric syndrome.

## 1. Introduction

The physical and psychological functions of many older adults are being negatively impacted by poor oral health. Difficulties with chewing, biting, swallowing, tasting, speaking, communicating, smiling, appearance, aesthetics, and self-esteem are common [1]. Among the frailest and most care-dependent older adults, dental caries, periodontal disease, tooth loss, and xerostomia are particularly prevalent [1,2,3,4]. Despite the fact that most chronic oral diseases are preventable and treatable, a variety of factors make it difficult to maintain good oral health as people age. This paper aims to present an overview of the published evidence on the systemic health factors that contribute to rapid oral health deterioration in older individuals, and to explore the implications of these factors for both general healthcare and oral healthcare provision.

## 2. Ageing

Ageing is typically viewed as a gradual decline in the functioning of various bodily systems, stemming from the accumulation of damaged tissue and substances caused by intrinsic or extrinsic mechanisms [5]. The process of biological ageing is a multifaceted and intricate phenomenon, and although the exact molecular mechanisms behind its onset and progression remain unclear, ample evidence suggests that oxidative stress may play a significant role [6]. Kinases, phosphatases, and transcription factors are particularly sensitive to changes in cellular redox status, and chronic or severe disruptions in this homeostasis can result in cell death or proliferation. Immune senescence, or the quantitative and qualitative changes in the immune system that accompany ageing, is another hallmark of this process. While immune senescence does not necessarily entail a progressive decline in immune function, it often leads to cytokine dysregulation, which can cause a chronic, low-grade inflammatory state. This inflammation may serve as a biological foundation for ageing and contribute to the onset of age-related diseases, increasing the risk of multimorbidity and mortality [6,7,8,9].

## 3. Ageing and Telomere Length

Telomere length is considered a useful biomarker of cellular ageing, as it reflects the repeated sequences of nucleotides that protect the ends of chromosomes [10]. With each replication of cells, telomeres shorten due to incomplete lagging strand replication, leading to cellular senescence once they reach a critically short length [11]. Studies have suggested that telomere length is sensitive to inflammation, as higher rates of telomere loss have been observed in a pro-inflammatory environment with increased blood cell replication [12]. This has prompted some researchers to explore the relationship between periodontal disease and telomere length [13,14,15,16]. In a NHANES study involving 21,000 participants aged 35–75 years, a significant correlation was found between periodontal disease and telomere length, particularly among women, overweight or obese individuals, and those with cardiometabolic comorbidities [17].

## 4. Diseases and Oral Health

Several studies have suggested a strong link between noncommunicable diseases and oral health, with demonstrated associations with oral diseases for various conditions including cancer, diabetes, cardiovascular diseases, depression, neurodegenerative conditions, rheumatic diseases, inflammatory bowel disease, gastric helicobacter pylori, obesity, and asthma [18]. The connection between oral health and these diseases is largely attributed to inflammation, although there are two other pathways that may explain the association [19,20,21]. Firstly, some systemic diseases have direct links to negative impacts on oral health and oral health-related quality of life (OHRQoL), such as Crohn’s disease [22,23], Beçhet’s disease [24,25,26], scleroderma [27,28], oral cancer [29,30,31,32], head and neck cancer [33], and Sjögren’s syndrome [34,35,36,37]. Secondly, some chronic diseases may indirectly affect oral health, as they can lead to reduced motivation regarding oral hygiene and care. For example, psychiatric [38,39,40,41,42,43,44,45,46,47] and neurological diseases [48,49,50,51,52,53], as well as Alzheimer’s disease [54,55], rheumatic [56], oncological [57], and cardiovascular diseases [58,59,60,61,62,63], can all have an impact.

Early diagnosis and treatment of oral conditions among older people with chronic diseases could prevent weak oral health and a decline in OHRQoL. However, individuals with cognitive disorders and those receiving palliative care may lose their ability to communicate their oral health needs, leading to under-reporting and underestimation of oral conditions [64]. This could result in healthcare providers failing to fully appreciate the extent of the problem, leading to untreated oral conditions and prolonged discomfort among these patients.

## 5. Multimorbidity and Polypharmacy

In 2013, a group of European researchers established a definition for multimorbidity, which refers to any combination of chronic disease with at least one other disease (acute or chronic), bio-psychosocial factor (associated or not), or somatic risk factor. This definition recognizes that any bio-psychosocial factor, somatic risk factor, social network, burden of diseases, healthcare consumption, and patient coping strategies may modify the effects of the multimorbidity. Multimorbidity can lead to increased disability, decreased quality of life, or frailty. While the concept of multimorbidity has been recognized and enhanced by European general practitioners [65], studies on its prevalence have not yet been conducted.

In populations of older adults with multimorbidities, the use of multiple medications is common, a phenomenon referred to as polypharmacy. Polypharmacy is associated with adverse outcomes, including medication–medication interactions, medication–disease interactions, decreased renal and hepatic function, and reduced lean body mass, hearing, vision, cognition, and mobility [66]. A meta-analysis showed that 38% of community-dwelling adults aged 60 years and older use five or more medications daily [67]. Additionally, almost half of care home residents are exposed to potentially inappropriate medications [66]. A systematic review identified 138 definitions of polypharmacy, but a numerical definition alone is insufficient to assess the safety and appropriateness of medication use. Therefore, a shift towards the term “appropriate polypharmacy”, using a holistic approach that considers comorbidities present, is needed [68].

## 6. Frailty and Oral Health

**Figure 1:**
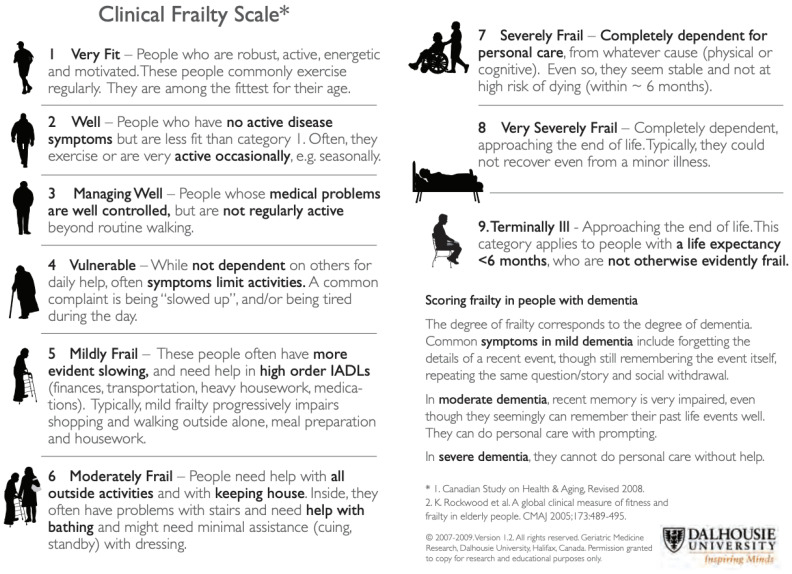
Clinical Frailty Scale (Dalhousie University, Halifax, NS, Canada), used with permission [69,70].

The concept of frailty has become increasingly important in recent decades, but a consensus on its definition has not yet been reached. There are two main approaches to defining frailty: one that focuses solely on physical functioning, and another that takes into account other domains, such as memory and mood. For example, the Fried frailty phenotype considers unintentional weight loss, self-reported exhaustion, physical activity, hand grip strength, and walking speed, while the multidimensional frailty index by Rockwood et al. also considers cognitive and psychological factors (Figure 1) [69,70]. The prevalence of frailty varies depending on the approach used, with higher rates found for multidimensional assessments [71].

A systematic review investigated the link between oral health and frailty, focusing on five longitudinal studies that used Fried’s frailty phenotype. These studies found that the number of teeth, oral functions, accumulation of oral health problems, and dry mouth symptoms were significantly associated with the incidence of frailty [72]. In community-dwelling older adults, oral pain was associated with weight loss and weak handgrip, while chewing problems were associated with low physical activity and low gait speed. Those who required dental prostheses were more likely to be prefrail or frail than others [73]. Further research is needed to determine whether oral health indicators can be used to assess frailty.

## 7. Sarcopenia and Oral Health

**Figure 2:**
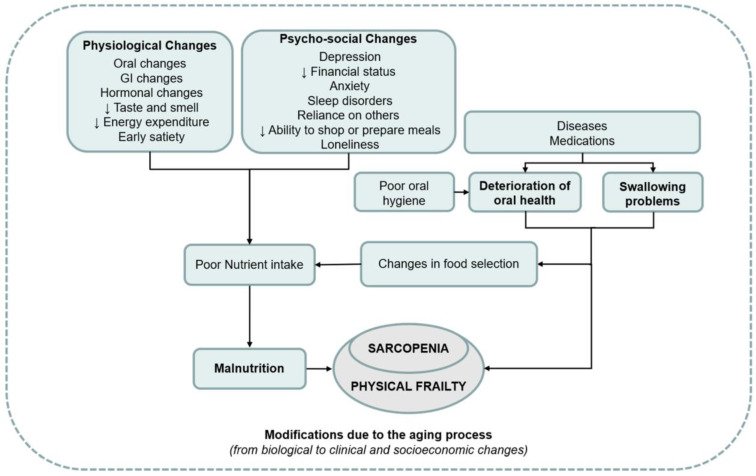
Overview of the associations between weak oral health, malnutrition, and sarcopenia, used with permission [79].

Sarcopenia is a condition that affects older individuals and causes a decline in muscle mass and strength. The prevalence of sarcopenia varies widely, between 3.2% to 40%, with the highest incidence in people above the age of 80 and those living in institutions [69,70,71,72,73,74]. Several risk factors have been identified, including age, chronic diseases, and physical activity levels. Chronic obstructive pulmonary disease, diabetes mellitus, and hypertension are among the chronic diseases that have been linked to sarcopenia [75]. Although it is a common issue in older adults, sarcopenia can be managed and even prevented with appropriate exercise and nutrition [74,76,77]. Interestingly, sarcopenia can also affect oral health in ways that are not well-known. As muscle mass declines, individuals may experience weaker temporomandibular and orofacial muscles, resulting in difficulty chewing and swallowing [78]. Figure 2 presents an overview of the associations between weak oral health, malnutrition, and sarcopenia [79].

## 8. Disability and Oral Health

Among older adults, there are bidirectional associations between oral health and disability, where both health outcomes can impact each other. Tooth loss, for instance, may lead to disabilities such as limitations in activities of daily living, instrumental activities of daily living, and mobility [80,81,82,83,84]. Conversely, disability may be associated with chronic illnesses, weak oral health, and reduced quality of life among older people [85]. The World Health Organization defines disability as an impairment that may be physical, cognitive, mental, sensory, emotional, developmental, or a combination of these, and which may occur during a person’s lifetime or from birth. Disabilities can cause physical and cognitive impairments, activity limitations, and participation restrictions [86]. To prevent disability among older adults, self-efficacy must be improved, and physical activity must be promoted [87]. Physical and cognitive functioning of older individuals can be assessed using several assessment instruments [88]. It is possible to analyze the order in which age-related declines occur by examining individuals and groups that are ageing physically and cognitively at different rates [89,90].

## 9. Impact of Ageing and Age-Related Diseases on General Healthcare Provision

There is a new trend in healthcare provision for older people which focuses on preventing premature admission to care homes. This trend offers various healthcare options, including the use of mobility aids, assistive technology devices, domiciliary healthcare, respite care, and telecare. By using assistive technology, the rate of functional decline in frail older people can be slowed down, while domiciliary healthcare aims to maximize independence, self-esteem, self-image, and quality of life [91,92]. Evidence suggests that domiciliary healthcare has positive outcomes, including improved quality of life, functional status, and reduced costs [93]. Informal care provision through visiting nurses, hospice carers, and physical therapists can also help older people live at home for a longer period. Respite care, which offers temporary relief to informal carers, has shown some positive effects, but more research is needed to support this claim [94,95,96].

Telecare, which involves the use of personal and environmental sensors in older people’s homes, has been available for several decades. New options include sensors for falls, epilepsy, enuresis, and security monitoring for temperature, carbon monoxide, and smoke detection. Although the benefits of telecare are not yet fully understood due to limited research data, it presents an opportunity to identify what works best for each individual and in which circumstances [97]. Despite the new healthcare options, informal carers, such as spouses, children, relatives, and friends, still have to provide much of the domiciliary care to frail older people.

## 10. Impact of Multimorbidity and Polypharmacy on Oral Healthcare Provision

Multimorbidity can lead to a range of physical and psychosocial issues in older adults. The complexity of this condition means that symptoms may be difficult to diagnose, and diseases may be masked or exacerbated by other health problems. In addition, treatment of one disease may be affected by the presence of other diseases. This can result in a gradual decline in overall health. Oral healthcare providers who work with older adults should have a thorough understanding of geriatrics and pharmacology, and collaborate closely with physicians and pharmacists to provide individualized care [98]. Older adults are particularly susceptible to adverse reactions to medication due to age-related changes in pharmacokinetics and pharmacodynamics, as well as the prevalence of polypharmacy [99,100,101]. Many medications can cause a decrease in saliva secretion rate, leading to dry mouth and a range of oral health problems [100]. Oral healthcare providers should consider the impact of medication on oral health and be cautious in prescribing medication to patients with polypharmacy. The modified Summated Xerostomia Inventory (Table 1) can be used to assess xerostomia. Practical treatments are available to alleviate the symptoms of dry mouth and improve overall physical and psychosocial well-being [102,103].

## 11. Impact of Frailty, Disability, and Care Dependency on Oral Healthcare Provision 

It is crucial for both formal and informal caregivers of older adults to understand that those who are frail or disabled are at significant risk of developing oral health problems. Caregivers should therefore take the responsibility of organizing a consultation with an oral healthcare provider. On the other hand, sudden deterioration of oral health in older individuals can be an early indicator of frailty and should prompt oral healthcare providers to arrange a consultation with a physician or geriatrician. Multiple epidemiological studies suggest that professional oral healthcare is urgently needed to address the unmet needs of older adults. To improve oral healthcare provision, there should be integration of oral healthcare into general healthcare, community programs that promote healthy behaviors, and access to preventive oral healthcare [104]. A crucial strategy is the development and implementation of an oral healthcare guideline to cater to older adults living in the community. As older adults prefer to age in place, new options for oral healthcare provision such as domiciliary oral healthcare, customised oral hygiene care aids, visiting dental hygienists and nurses, and oral hygiene telecare should be developed. Unfortunately, not all oral healthcare offices are easily accessible for older adults who are frail, disabled, or care dependent. Therefore, it is the responsibility of oral healthcare providers to make their premises easily and safely accessible for this group of individuals. Only when oral healthcare providers accept and face this responsibility can dentistry be transformed into medical oral healthcare and dentists be upgraded to oral physicians.

## 12. Epilogue

The risk of rapid deterioration in oral health is heightened by ageing, age-related diseases, multimorbidity, polypharmacy, frailty, disability, sarcopenia, and inappropriate oral hygiene care. Providing oral care to frail older people can be complex due to additional factors such as lifestyle (nutrition and smoking habits) and the motivation of the patient or caregivers to achieve adequate oral hygiene. Depending on the degree of frailty, the living environment, older people’s preferences regarding oral health (shared decision making), and life expectancy, an individualized oral care plan must be made, in which the goals with regard to oral health are outlined. These goals should be formulated as concretely and measurably as possible and be realistic and acceptable to all parties, whereas the principles of palliative care should often be used for frail older people. 

Furthermore, physicians should also be aware of the potential impact of risk factors of rapid deterioration on the oral health of their patients. Physicians should pay attention to their patients’ oral health when developing diagnosis and treatment plans. Furthermore, physicians should be alert to the side effects of the medications they prescribe, as some medications can negatively impact oral health. They may consider prescribing alternative medications with less harmful effects on oral health. For example, medication with a negative effect on saliva secretion rates as a side-effect, can be replaced by other medication with a less xerogenic effect.

In general, it is important for physicians and dentists to view oral health as an integral part of a patient’s overall health. By working together and providing proactive care, they can reduce the negative impact of these factors on oral health and improve patients’ quality of life. Finally, it is crucial for all care providers to raise awareness about the importance of maintaining good oral health and the consequences of neglecting it, in order to prevent weakened oral health from becoming a geriatric syndrome [4].

## Figures and Tables

**Table 1:** Modified summated xerostomia inventory.

		Never	Occasionally	Often
1	My mouth feels dry when eating a meal	1	2	3
2	My mouth feels dry	1	2	3
3	I have difficulty in eating dry foods	1	2	3
4	I have difficulties swallowing certain foods	1	2	3
5	My lips feel dry	1	2	3

Summated score < 8 no xerostomia; summated score ≥ 8 xerostomia.

## Data Availability

Not applicable.
